# Dietary supplementation with *Clostridium autoethanogenum* protein improves growth performance and promotes muscle protein synthesis by activating the mTOR signaling pathway of the broiler

**DOI:** 10.3389/fvets.2024.1389738

**Published:** 2024-06-11

**Authors:** Chunqiao Shan, Yan Liu, Chaoxin Ma, Chuang Li, Qiuchen Liu, Sisi Liu, Guotuo Jiang, Jing Tian

**Affiliations:** ^1^School of Biological Engineering, Dalian Polytechnic University, Dalian, China; ^2^Dalian Sanyi Biotechnology Research Institute, Dalian Sanyi Animal Medicine Co., Ltd., Dalian Liaoning, China; ^3^Research Quality Control Center, Jiangsu Sanyi Animal Nutrition Technology Co., Ltd., Xuzhou, China; ^4^Harbin Academy of Agricultural Sciences, Harbin Heilongjiang, China

**Keywords:** *Clostridium autoethanogenum* protein, broiler, growth performance, mTOR, antioxidant activity, immune function

## Abstract

The experiment aimed to evaluate the effects of different ratios of *Clostridium autoethanogenum* protein (CAP) used in the diets on the growth performance, muscle quality, serum indexes, and mTOR pathway of white feather broilers. Four hundred and eighty 1-day-old Arbor Acres (AA) broilers, comprising equal numbers of males and females, were randomly assigned to one of four treatments, and each treatment consisted of 12 replicates of 10 birds. Four diets were formulated based on isoenergetic and isonitrogenous principles. The control group (CAP 0) did not receive any CAP, while the experimental groups received 2% (CAP 2), 3% (CAP 3), and 4% (CAP 4) of CAP for six weeks. Compared with the CAP0, (1) The feed conversion ratio (FCR) was lower (*p* < 0.05), and the leg muscle yield was higher (*p* < 0.05) in the CAP3 and CAP4; (2) The serum levels of TP, ALB, T-AOC, and SOD were improved in the CAP3 (*p* < 0.05); (3) The expression of *Lipin-1* gene was down-regulated and *AMPKɑ2*, *Akt*, and *4E-BP1* genes were up-regulated in the experiment group (*p* < 0.05); (4) The inclusion of 3% CAP in the diet increased the levels of 4E-BP1, S6K1, Akt, and AMPKɑ2 phosphorylation by modulating the mTOR signaling pathway (*p* < 0.05). In conclusion, broiler diets containing 3% CAP can activate the mTOR signaling pathway to promote muscle synthesis and improve growth performance.

## Introduction

The world’s population has increased dramatically in recent years and could reach 10 billion by 2050 (1, 2). As a result, the global demand for meat is expected to exceed 400 million tons, while the demand for dairy products will surpass 800 million tons (3). Soybean meal is widely used in the breeding industry because of its high protein content and relatively low antinutritional factors. However, China relies heavily on soybean imports and unstable prices, severely limiting the growth of the animal husbandry industry. Therefore, reducing the use of crude protein (CP) in diets and developing and using new protein sources are essential to alleviate the shortage of protein resources.

Low protein diets are based on the ideal protein model by adding certain synthetic amino acids to compensate for the reduction in protein so that the type, amount, and ratio of amino acids required for animal growth are met, which can improve feed conversion, animal nitrogen deposition, and emissions while ensuring that the normal production performance of livestock and poultry is maintained (4–6). Compared with traditional rations, low-protein diets can reduce feeding costs, improve animal health, and have great advantages in the area of environmentally friendly and healthy breeding (7, 8). Supported by national policies, low-protein diets have been widely used in the breeding industry of China.

Microorganisms such as yeasts, algae, fungi, and bacteria can produce single-cell proteins (SCPs) that contain high amounts of protein, vitamins and minerals. SCP can effectively substitute protein feedstocks like fishmeal and soybean meal (9, 10). In addition, the production of SCP is not limited by time and geography and can be factory-produced on a large scale (11). Numerous studies have confirmed that SCP can be used in broiler diets to reduce the need for soybean meal. Furthermore, the appropriate amount of SCP can increase the breast and leg muscle yields while also improving the weight gain, FCR, and economic efficiency of broilers (12–14).

CAP produced by *Clostridium autoethanogenum* (CA) is a SCP with a relatively balanced amino acid profile (15, 16). It has potential as a replacement for protein raw materials in livestock, poultry, and aquatic products. Research has demonstrated that CAP can serve as a viable replacement for fishmeal due to its high protein and amino acid content, lack of anti-nutritional factors, and low biogenic amine content (17, 18). Besides, CA can convert inorganic matter into organic matter and decrease the discharge of industrial waste, making it a valuable contributor to the promotion of environmentally friendly and sustainable economic development (19).

The mTOR pathway is responsible for nutrient sensing and regulates organismal growth and metabolism (20–22). Inhibiting the mTOR pathway reduces protein synthesis while activating it promotes protein synthesis (23, 24). Additionally, it promotes cell growth and proliferation by increasing protein and lipid synthesis and decreasing cellular autophagy (25). Numerous studies in aquaculture have demonstrated that CAP can regulate the mTOR signaling pathway, improve organismal growth, and enhance growth performance (26–28). However, there is a lack of research on CAP in livestock and poultry, and the mechanisms of metabolism and growth regulation remain unclear. Therefore, studying the effects and regulatory mechanisms of CAP feeding in broilers can provide theoretical support for the application of CAP in broilers, which is of great research value and theoretical significance. We hypothesized that the appropriate proportions of CAP could regulate broiler growth through the mTOR signaling pathway. Therefore, a feeding trial was conducted using four gradients of CAP (CAP0, CAP2, CAP3, and CAP4) to assess its effects on broiler performance, muscle quality, serum indexes, and the mTOR pathway.

## Materials and methods

### Experimental design and diets

In our experiment, a total of 480 one-day-old Arbor Acres (AA) broilers (initial body weight 43.84 ± 1.38 g) purchased from Zhenghe Poultry Industry Co., Ltd. in Shangqiu City were randomly divided into four treatment groups, with 12 replicates of 10 birds per treatment (half male and half female). The broilers were fed with four isoenergetic and isonitrogenous diets containing CAP at 0 g/kg (Control, CAP0), 20 g/kg (CAP2), 30 g/kg (CAP3), and 40 g/kg (CAP4) respectively, and the experimental feed formulation complied with DB22_T3207-2020 “Technical specifications for low protein diet for broiler chickens.” The experiment consisted of two stages. The early-stage diet was fed to the subjects from 1 to 21 days, while the later-stage diet was fed from 22 to 42 days. For the nutritional composition and ingredients of the diets, refer to Tables 1, 2. The temperature was kept at 33°C for the initial three days, after which it was gradually decreased by 1°C every two days until it reached 20°C. The light period consisted of three consecutive days after hatching, followed by 23 h of light and 1 h of darkness throughout the remainder experimental period. The broilers were vaccinated according to the immunization schedule and had free access to feed and fresh water, while the relative humidity was maintained at 60%.

**Table 1:** Composition and nutrient content of broiler diets from 1 to 21 d (Air dried basis) %.

Items	Groups^①^			
	CAP0	CAP2	CAP3	CAP4
*Ingredients, %*				
Corn	62.12	62.45	61.95	62.28
Wheat Bran	0.00	2.90	5.00	6.20
Soybean meal	28.00	24.80	22.73	21.20
Expanded soybean	5.00	3.00	2.50	1.50
CAP^②^	0.00	2.00	3.00	4.00
Soybean oil	1.00	1.00	1.00	1.00
CaHPO_4_·2H_2_O	1.50	1.55	1.52	1.53
Stone powder	1.38	1.37	1.41	1.43
NaCl	0.30	0.30	0.30	0.30
Mineral premix^③^	0.10	0.10	0.10	0.10
Vitamin premix^④^	0.03	0.03	0.03	0.03
Phytase	0.02	0.02	0.02	0.02
Choline chloride	0.10	0.10	0.10	0.10
Lysine	0.18	0.14	0.12	0.10
Methionine	0.20	0.17	0.16	0.15
Threonine	0.07	0.07	0.06	0.06
Total	100	100	100	100
*Nutrient levels*				
Crude protein, %	19.56	19.55	19.56	19.57
*CF*, %	2.94	2.89	2.90	2.87
Ash, %	4.45	5.48	4.15	4.01
ME, Kcal/kg^⑤^	2,973	2,973	2,972	2,974
Calcium, %	0.92	0.91	0.91	0.92
P-total, %	0.62	0.62	0.62	0.62
Lysine, %	1.15	1.15	1.15	1.15
Methionine, %	0.50	0.50	0.50	0.50
Threonine, %	0.80	0.80	0.80	0.80
tryptophan, %	0.22	0.21	0.21	0.20
Valine, %	0.90	0.93	0.94	0.96

① CAP0: 0 g of CAP per kg of diet; CAP2: 20 g of CAP per kg of diet; CAP3: 30 g of CAP per kg of diet; CAP4: 40 g of CAP per kg of diet.

② CAP: *Clostridium autoethanogenum* protein.

③ The mineral premix contains Cu 8 mg, Fe 80 mg, Mn80 mg, Zn 80 mg, I 0.5 mg, Se 0.3 mg/kg of diet.

④ The vitamin premix contains VA 8000 IU, VD_3_ 1,000 IU, VE 20 IU, VK_3_ 0.5 mg, VB_1_ 2 mg, VB_2_ 8 mg, VB_6_ 3 mg, VB_12_ 0.01 mg, niacin 35 mg, folate 0.55 mg, pantothenic acid 10 mg, biotin 0.15 mg.

⑤ ME: metabolizable energy. The nutrient level is the calculated value.

**Table 2:** Composition and nutrient content of broiler diets from 22–42 d (Air dried basis) %.

Items	Groups^①^			
	CAP0	CAP2	CAP3	CAP4
*Ingredients, %*				
Corn	67.60	68.20	67.19	66.13
Wheat Bran	0.00	2.65	5.30	8.07
Soybean meal	23.43	20.53	17.90	15.20
Expanded soybean	3.30	1.00	1.00	1.00
CAP^②^	0.00	2.00	3.00	4.00
Soybean oil	2.00	2.00	2.00	2.00
CaHPO_4_·2H_2_O	1.20	1.20	1.20	1.20
Stone powder	1.43	1.45	1.47	1.47
NaCl	0.30	0.30	0.30	0.30
Mineral premix^③^	0.10	0.10	0.10	0.10
Vitamin premix^④^	0.03	0.03	0.03	0.03
Phytase	0.02	0.02	0.02	0.02
Choline chloride	0.10	0.10	0.10	0.10
Lysine	0.18	0.13	0.11	0.10
Methionine	0.18	0.16	0.15	0.14
Threonine	0.12	0.11	0.11	0.11
Tryptophan	0.01	0.02	0.02	0.03
Total	100	100	100	100
Nutrient levels				
Crude protein, %	17.5	17.5	17.5	17.5
*CF*, %	2.67	2.61	2.65	2.69
Ash, %	4.97	5.02	5.06	5.1
ME, Kcal/kg^⑤^	3,062	3,062	3,062	3,062
Calcium, %	0.85	0.85	0.85	0.85
P-total, %	0.55	0.54	0.54	0.55
Lysine, %	1.00	1.00	1.00	1.00
Methionine, %	0.46	0.46	0.46	0.46
Threonine, %	0.76	0.76	0.76	0.76
tryptophan, %	0.20	0.20	0.20	0.20
Valine, %	0.80	0.82	0.84	0.85

① CAP0: 0 g of CAP per kg of diet; CAP2: 20 g of CAP per kg of diet; CAP3: 30 g of CAP per kg of diet; CAP4: 40 g of CAP per kg of diet.

② CAP: *Clostridium autoethanogenum* protein.

③ The mineral premix contains Cu 8 mg, Fe 80 mg, Mn 80 mg, Zn 80 mg, I 0.5 mg, Se 0.3 mg/kg of diet.

④ The vitamin premix contains VA 8000 IU, VD_3_ 1,000 IU, VE 20 IU, VK_3_ 0.5 mg, VB_1_ 2 mg, VB_2_ 8 mg, VB_6_ 3 mg, VB_12_ 0.01 mg, niacin 35 mg, folate 0.55 mg, pantothenic acid 10 mg, biotin 0.15 mg.

⑤ ME: metabolizable energy. The nutrient level is the calculated value.

### Production performance

At the end of 1, 21, and 42 d, chickens (after a 12 h fast) were weighed, and feed intake and quantity of dead broiler were recorded for each treatment pen for the calculation of average daily gain (ADG), average daily feed intake (ADFI), and FCR. At 42 d, blood samples were collected from 24 broilers (Randomly selected 6 chickens for each treatment, male and female halves) via bleeding from the jugular vein. After obtaining the blood sample, it was centrifuged at 3,000 g at 4°C for 10 min. In this way, the serum is obtained. Following the jugular vein bloodletting and execution, the slaughtering performance was evaluated by the NYT823-2020 standard.

### Muscle composition

Breast muscle samples were baked in an oven at 65°C to make air-dried samples, and then analyzed according to the (29) standard method. Briefly, the samples were dried in an oven at 105°C until constant weight for calculation of dry matter. The CP content was determined by the Kjeldahl nitrogen method. The crude lipid content was measured by ether extraction using a Soxhlet extractor. The ash content was determined by combustion at 550°C in a muffle furnace for 6 h.

### Serum biochemical and antioxidant indexes

Alanine aminotransferase (ALT), aspartate transaminase (AST), alkaline phosphatase (ALP), lactate dehydrogenase (LDH), amylase (AMS), total protein (TP), albumin (ALB), uric acid (UA), glucose (GLU), triglyceride (TG), total cholesterol (TC), high-density lipoprotein (HDL), low-density lipoprotein (LDL), total antioxidant capacity (T-AOC), catalase (CAT), malondialdehyde (MDA), glutathione peroxidase (GSH-Px), and superoxide dismutase (SOD) were determined by assay kits (Nanjing Jiancheng Co., Nanjing, China).

### Determination of immunological indexes

Procalcitonin (PCT), immunoglobulin A (IgA), immunoglobulin G (IgG), interleukin-1 (IL-1), interleukin-6 (IL-6), and interleukin-8 (IL-8) were determined by assay kits (Shanghai Enzyme Link Biotechnology Co., Shanghai, China).

### 2.6 RNA extraction, cDNA synthesis, and real-time PCR

RNA was first extracted from breast muscle and liver samples using the RNAiso Plus kit (Takara, Japan). The concentrations and OD260/OD280 of RNA were then assessed using a Micro-Drop spectrophotometer (BIO-DL, United States), and complementary DNA (cDNA) was synthesized using a PrimeScript™ RT reagent kit (Takara, Japan). The reaction system was prepared according to the fluorescent quantitative PCR kit. The following were the thermocycling conditions for the target genes: 95°C for 30 s, 95°C for 15 s, and 60°C for 30 s to collect the fluorescence signals, and 40 cycles were performed. Quantification of relative gene expression was performed using 2^-ΔΔCT^. The targeted primers for several genes in this study are indicated in Table 3.

**Table 3:** Primer sequences.

Gene	Primer sequence(5′-3′)	Accession number
mTOR	F:CTACGCGCCATTGTATTTGC	XM_417614.8
	R:AGCCATTCCAGAGCACGTTT	
LIPIN-1	F:ATGGGAGTTCTGCGTTCCAG	XM_004935771.5
	R:TGCCCTGTGTAATCTATGGGC	
S6K1	F:CATGATTTCCAAACGACCAGA	NM_001030721.2
	R:AGTAAACCAAACAAGCCCTCC	
PI3K	F:TCGCCACAACAGTAACATCA	NM_001004410.2
	R:ACAAAGGGCACACGCTCT	
IGF-1	F:AGCAGTAGACGCTTACACCA	NM_001004384.3
	R:CACAGTACATCTCCAGCCTCC	
AMPKɑ2	F:AGCTGTGTCGTGCAGTACC	NM_001039605.2
	R:TTGCCGAAGGTGCCGAC	
AKT	F:CTGCCGTGAGCCCAGTTAG	NM_205055.2
	R:TCAGCTACTTATGGCTGCGG	
4EBP1	F:ACCAGGATTATTTATGACCG	XM_424384
	R:TTCACCTACATTCGCTTTCT	

mTOR, mechanistic target of rapamycin kinase; LIPIN-1, lipin 1; S6K1, Ribosomal protein S6 Kinase 1; PI3K, Phosphoinositide 3-kinase; IGF-1, Insulin growth factor-1; AMPKɑ2, adenosine monophosphate-activated protein kinase ɑ2; AKT, threonine kinase/protein B; 4EBP1, Eukaryotic initiation factor 4E binding protein-1.

### 2.7 Protein extraction and Western blot

According to the mass and volume of each sample, add the corresponding volume of lysate, let it stand for 5 min, centrifuge at 12,000 r for 10 min, and the supernatant is the protein extract. The protein concentration of the samples was determined according to the instructions of the BCA Protein Concentration Assay Kit (Solarbio Beijing, China). After determining the quantity of protein to be loaded, diluted protein samples with 5 × Loading Buffer and PBS and boiled at 100°C for 5 min. Equal amounts of protein were loaded into the wells of the SDS-PAGE gel, and the proteins were then transferred from the gel to the PVDF membrane. Subsequently, the membrane was closed with 5% skimmed milk powder for an hour. After incubation with primary and secondary antibodies, the PVDF membrane was placed in a chemical exposure apparatus, evenly sprinkled with ECL chemiluminescent solution, and the exposure time was set to expose the membrane. The results were analyzed using a gel image processing system (ImageJ) to analyze the gray scale values of the target bands. The antibodies used in the present study included: phospho-mTOR (Ser2448), mTOR, 4EBP1, AKT, and phospho-AKT (Ser473) purchased from Proteintech Group; phospho-p70S6 Kinase (Thr389), AMPK alpha-2, and phospho-AMPK alpha-1,2 (Thr172, Thr183) purchased from Thermo Fisher Scientific; p70S6 Kinase purchased from Affinity; phospho-4E-BP1 (Thr37/46) (236B4) purchased from cell signaling Technology.

### Statistical analysis

The experimental data was collated using Excel 2021, and the Shapiro–Wilk test was performed, followed by one-way ANOVA (one-way ANOVA) using SPSS 26.0 and Duncan’s method for multiple comparisons. The values were presented as mean ± SEM, and differences were considered statistically significant at *p <* 0.05.

## Results

### Effects of using CAP in diets on broiler growth performance

The effects of different proportions of CAP added to the diets on the growth performance of broilers are shown in Table 4. At 22–42 d, the FCR was lower in the CAP3 group than in the CAP0 group (*p* < 0.05). At the 1–42 d, the FCR was lower in the CAP3 and CAP4 groups than in the CAP0 group (*p* < 0.05).

**Table 4:** Effects of using CAP in diets on broiler growth performance.

Items^②^	Groups^①^				SEM^③^	*p*
	CAP0	CAP2	CAP3	CAP4		
*BW (g)*						
1 d	43.88	43.92	43.86	43.68	0.20	0.979
21 d	759.03	805.79	805.50	791.06	8.57	0.176
42 d	2175.89	2207.81	2261.83	2253.65	13.32	0.071
*ADG (g/d)*						
1–21 d	34.05	36.28	36.27	35.59	0.41	0.179
22–42 d	67.47	66.76	69.35	69.65	0.55	0.182
1-42d	50.76	51.52	52.81	52.62	0.32	0.070
*ADFI (g/d)*						
1–21 d	47.89	49.45	49.57	48.96	0.46	0.580
22–42 d	137.61	135.49	133.75	136.98	0.74	0.264
1–42 d	92.75	92.47	91.66	92.97	0.43	0.733
*FCR*						
1–21 d	1.41	1.37	1.37	1.38	0.01	0.058
22–42 d	2.04^a^	2.03^a^	1.93^b^	1.97 ^ab^	0.01	0.010
1–42 d	1.83^a^	1.80^ab^	1.74^c^	1.77^bc^	0.01	0.001

① CAP0: 0 g of CAP per kg of diet; CAP2: 20 g of CAP per kg of diet; CAP3: 30 g of CAP per kg of diet; CAP4: 40 g of CAP per kg of diet.

② BW, body weight; ADG, average daily gain; ADFI, average daily feed intake; FCR, feed conversion ratio.

③ SEM, standard error of the mean.

^a-c^Means with different superscripts in the same row are significantly different.

### Effects of using CAP in diets on the slaughtering performance of broiler chickens

As shown in Table 5, CAP had no significant effect on dressing percentage, percentage of half-eviscerated yield, and percentage of eviscerated yield (*p >* 0.05) but had an impact on breast muscle yield and leg muscle yield (*p <* 0.05). The breast muscle yield of the CAP3 group was higher than the other groups (*p <* 0.05), and the leg muscle yield of the CAP0 group was lower than the CAP3 and CAP4 groups (*p <* 0.05).

**Table 5:** Effects of using CAP in diets on broiler slaughter performance.

Items	Group^①^				SEM^②^	*p*
	CAP0	CAP2	CAP3	CAP4		
Dressing percentage %	93.28	92.24	93.14	92.09	0.31	0.446
Percentage of half-eviscerated yield %	86.25	84.96	85.93	85.27	0.27	0.336
Percentage of eviscerated yield %	73.43	73.57	74.64	73.78	0.31	0.551
Breast muscle yield %	24.49^b^	25.84^b^	27.78^a^	25.11^b^	0.37	0.004
Leg muscle yield %	18.90^b^	19.77^ab^	20.34^a^	20.40^a^	0.20	0.016

① CAP0: 0 g of CAP per kg of diet; CAP2: 20 g of CAP per kg of diet; CAP3: 30 g of CAP per kg of diet; CAP4: 40 g of CAP per kg of diet.

② SEM, standard error of the mean.

^a,b^Means with different superscripts in the same row are significantly different.

### Effects of using CAP in diets on muscle composition of broiler chickens

Table 6 describes the effects of adding CAP on meat quality and composition of broiler. The muscle crude protein content in the CAP0 group was lower than the CAP3 group (*p <* 0.05).

**Table 6:** Effects of using CAP in diets on muscle composition of broiler.

Items^②^	Groups^①^				^③^SEM	*p*
	CAP0	CAP2	CAP3	CAP4		
DM, %	88.12	88.66	88.69	89.14	0.18	0.292
CP, %	68.17^b^	69.29^ab^	71.09^a^	70.14^ab^	0.38	0.029
Ash, %	3.30	3.21	2.76	2.92	0.11	0.289
EE, %	13.10	12.71	11.90	12.66	0.20	0.197
OM, %	84.82	85.45	85.93	86.21	0.25	0.220

① CAP0: 0 g of CAP per kg of diet; CAP2: 20 g of CAP per kg of diet; CAP3: 30 g of CAP per kg of diet; CAP4: 40 g of CAP per kg of diet.

② DM, dry matter; CP, crude protein; Ash, crude ash; EE, ether extract; OM, organic matter.

③ SEM, standard error of the mean.

^a,b^Means with different superscripts in the same row are significantly different.

### Effects of using CAP in diets on serum biochemical indices of broiler chickens

As shown in Table 7, dietary CAP inclusion levels influenced the serum biochemical indexes of broilers. The CAP0 and CAP4 groups had lower TP levels than the CAP3 group (*p <* 0.05). Broilers fed with CAP0 and CAP2 diets had lower UA content compared to the CAP3 and CAP4 diets (*p <* 0.05). The CAP3 group had the lowest TG levels, followed by the CAP4 group. The AST activity in CAP4 was higher than CAP0 and CAP2 groups (*p <* 0.05).

**Table 7:** Effects of using CAP in diets on serum biochemical indexes of broilers.

Items^②^	Groups^①^				SEM^③^	*p*
	CAP0	CAP2	CAP3	CAP4		
GLU (mmol/L)	11.4	11.01	10.58	10.63	0.14	0.108
TP (g/L)	27.40^b^	28.35^ab^	29.32^a^	28.09^b^	0.23	0.02
AMS (U/L)	428.22	440.71	410.93	425.59	10.43	0.816
UA (μmol/L)	264.57^b^	267.79^b^	285.07^a^	295.65^a^	3.29	<0.001
TG (mmol/L)	0.39^a^	0.36^ab^	0.30^b^	0.32^b^	0.01	0.022
ALT (U/L)	5.02	5.32	4.76	5.04	0.19	0.813
AST (U/L)	263.67^b^	267.54^b^	281.44^ab^	308.05^a^	5.97	0.024
ALP (U/L)	2528.34^b^	2728.80^a^	2819.79^a^	2799.75^a^	33.38	0.002
LDH (U/L)	285.98	266.72	279.46	283.70	7.49	0.825
HDL (mmol/L)	2.21	2.15	2.33	2.09	0.06	0.599
LDL (mmol/L)	0.66	0.64	0.75	0.70	0.02	0.227
ALB (g/L)	14.64^b^	15.71^a^	16.25^a^	16.29^a^	0.2	0.003
TC (mmol/L)	3.61	3.33	3.53	3.75	0.06	0.096

① CAP0: 0 g of CAP per kg of diet; CAP2: 20 g of CAP per kg of diet; CAP3: 30 g of CAP per kg of diet; CAP4: 40 g of CAP per kg of diet.

② GLU, glucose; TP, total protein; AMS, amylase; UA, uric acid; TG, triglycerides; ALT, alanine aminotransferase; AST, aspartate transaminase; ALP, alkaline phosphatase; LDH, lactate dehydrogenase; HDL, high-density lipoprotein; LDL, low-density lipoprotein; ALB, albumin; TC, total cholesterol.

③ SEM, standard error of the mean.

^a,b^Means with different superscripts in the same row are significantly different.

### Effects of using CAP in diets on antioxidant indexes in broiler chickens

As shown in Table 8, the T-AOC in the CAP0 group was lower than in the CAP2 and CAP3 groups (*p <* 0.05). As for the GSH-Px, the CAP2, CAP3, and CAP4 groups were higher than the CAP0 group (*p <* 0.05). SOD was higher in the CAP3 group than in the CAP0 and CAP4 groups (*p <* 0.05).

**Table 8:** Effects of using CAP in diets on serum antioxidant indexes of broilers.

Items^②^	Groups^①^				SEM^③^	*p*
	CAP0	CAP2	CAP3	CAP4		
T-AOC (U/mL)	7.43^b^	9.28^a^	9.07^a^	8.49^ab^	0.27	0.048
CAT (U/mL)	8.35	8.22	8.55	8.23	0.22	0.955
MDA (nmol/mL)	2.71	2.45	2.37	2.43	0.09	0.616
GSH-Px (U/mL)	163.21^b^	176.50^a^	186.59^a^	189.73^a^	2.97	0.002
SOD (U/mL)	83.26^c^	94.63^ab^	99.36^a^	87.22^bc^	1.81	0.002

① CAP0: 0 g of CAP per kg of diet; CAP2: 20 g of CAP per kg of diet; CAP3: 30 g of CAP per kg of diet; CAP4: 40 g of CAP per kg of diet.

② T-AOC, total antioxidant capacity; CAT, catalase; MDA, malondialdehyde; GSH-Px, glutathione peroxidase; SOD, superoxide dismutase.

③ SEM, standard error of the mean.

^a,b^Means with different superscripts in the same row are significantly different.

### Effects of using CAP in diets on immune and inflammatory factors in broiler chickens

Table 9 shows the effects of adding CAP on the immune and inflammatory factors of the broiler. Broilers fed with CAP3 and CAP4 diets had significantly higher IgG levels compared to those fed with CAP0 and CAP2 diets (*p* < 0.05).

**Table 9:** Effects of using CAP in diets on serum immune and inflammatory factors in broilers.

Items^②^	Groups^①^				SEM^③^	*p*
	CAP 0	CAP 2	CAP 3	CAP 4		
PCT (pg/mL)	1408.15	1405.46	1473.96	1421.81	10.97	0.086
IgA (ug/mL)	376.73	370.98	364.54	361.83	2.57	0.164
IgG (ug/mL)	3170.69^b^	3274.48^b^	3534.82^a^	3521.08^a^	47.85	0.005
IL-1 (pg/mL)	245.49	263.27	254.11	273.13	5.22	0.284
IL-6 (pg/mL)	63.51	61.80	62.21	66.01	1.09	0.545
IL-8 (pg/mL)	394.52	393.7	388.52	395.06	3.03	0.881

① CAP0: 0 g of CAP per kg of diet; CAP2: 20 g of CAP per kg of diet; CAP3: 30 g of CAP per kg of diet; CAP4: 40 g of CAP per kg of diet.

② PCT, procalcitonin; IgA, immunoglobulin A; IgG, immunoglobulin G; IL-1, interleukin-1; IL-6, interleukin-6; IL-8, interleukin-8.

③ SEM, standard error of the mean.

^a,b^Means with different superscripts in the same row are significantly different.

### Gene expression associated with mammalian targets of the mTOR signaling pathway in breast muscle and liver

**Figure 1:**
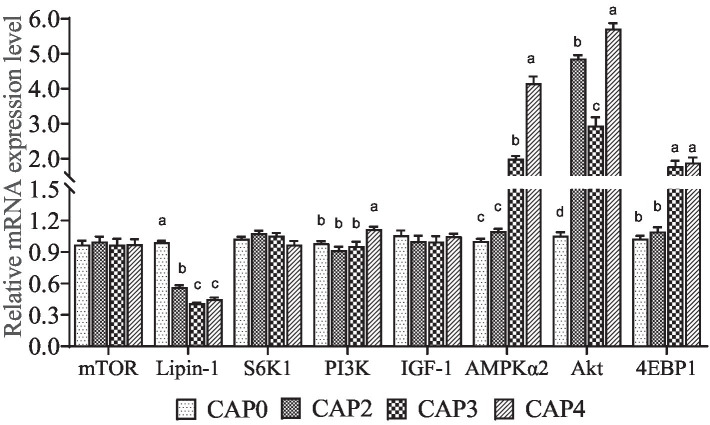
Effects of CAP on the expression pattern of key genes of the mTOR pathway in breast muscle. Data are presented as means and standard errors (±SE) (*n* = 6).

The mRNA levels of genes related to the mTOR signaling pathway in chest muscle are shown in Figure 1. The expression level of *Lipin-1* was lower in the CAP2, CAP3, and CAP4 groups compared to the CAP0 group, with the lowest level observed in the CAP3 group (*p <* 0.05). In contrast, *AKT* expression was higher in the CAP4 groups than in the CAP0 groups (*p <* 0.05). Broiler-fed diets CAP4 had higher expression of *PI3K* than those fed diet CAP0 (*p <* 0.05). Broilers fed CAP3 and CAP4 diets showed a higher mRNA level of *AMPKɑ2* and *4EBP1* compared with broiler-fed diets CAP0 and CAP2 (*p <* 0.05). Additionally, a difference was observed between the CAP3 and CAP4 groups in the *AMPKɑ2* gene (*p <* 0.05).

**Figure 2:**
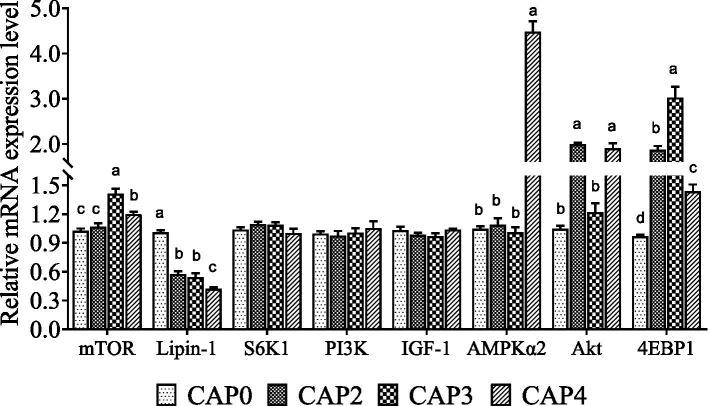
Effects of CAP on the expression pattern of key genes of the mTOR pathway in the liver. Data are presented as means and standard errors (±SE) (*n* = 6). Significant differences (*p* < 0.05) are indicated by different alphabetical superscripts above the bars, while the same letter indicates no difference.

The mRNA levels of genes related to the mTOR signaling pathway in the liver are shown in Figure 2. Broiler-fed diets CAP3 and CAP4 showed a higher mRNA level of *mTOR* than broiler-fed diets CAP0 and CAP2 (*p <* 0.05). Moreover, the CAP3 group was higher than the CAP4 (*p <* 0.05). The mRNA level of the *Lipin-1* in the CAP0 group was higher than the other groups (*p <* 0.05), and the CAP4 group had significantly higher expression of *AMPKɑ2* than the other groups (*p <* 0.05), which is consistent with the trend in the chest muscle. *Akt* relative mRNA expression level was higher in the groups CAP2 and CAP4 compared with the CAP0 and CAP3 groups (*p <* 0.05). *4E-BP1* expressions increased in treatment groups compared with the control group, and the CAP3 group was higher than the other groups (*p <* 0.05).

### Protein expression associated with mammalian targets of the mTOR signaling pathway in breast muscle and liver

**Figure 3:**
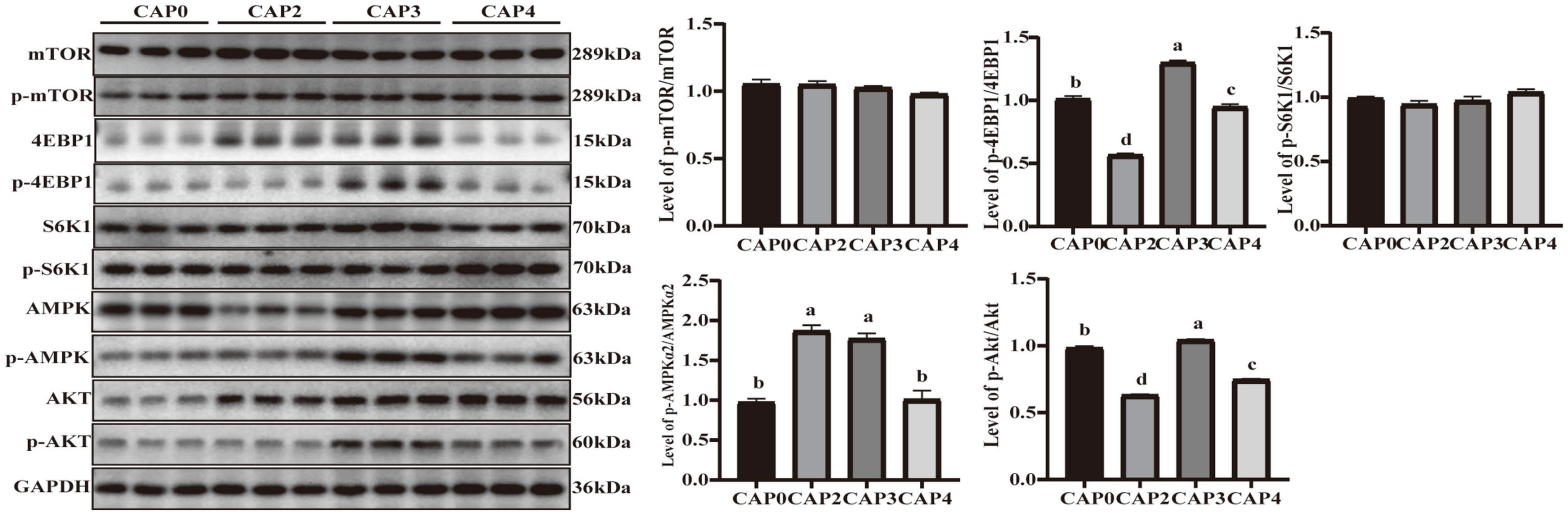
Protein expression levels and phosphorylation levels of some selected breast muscle genes involved in protein synthesis and energy metabolism in broilers fed CAP diets. Data are presented as means and standard errors (±SE) (*n* = 6). Significant differences (*p* < 0.05) are indicated by different alphabetical superscripts above the bars, while the same letter indicates no difference.

The present study evaluated the protein phosphorylation of key regulators in the mTOR signaling pathway in the breast muscle (Figure 3). The phosphorylation levels of 4E-BP1 and Akt showed the same trend and were higher in the CAP3 group than in the other groups (*p <* 0.05). Phosphorylation levels of AMPKɑ2 were higher in the CAP2 and CAP3 groups compared to the control group (*p <* 0.05).

**Figure 4:**
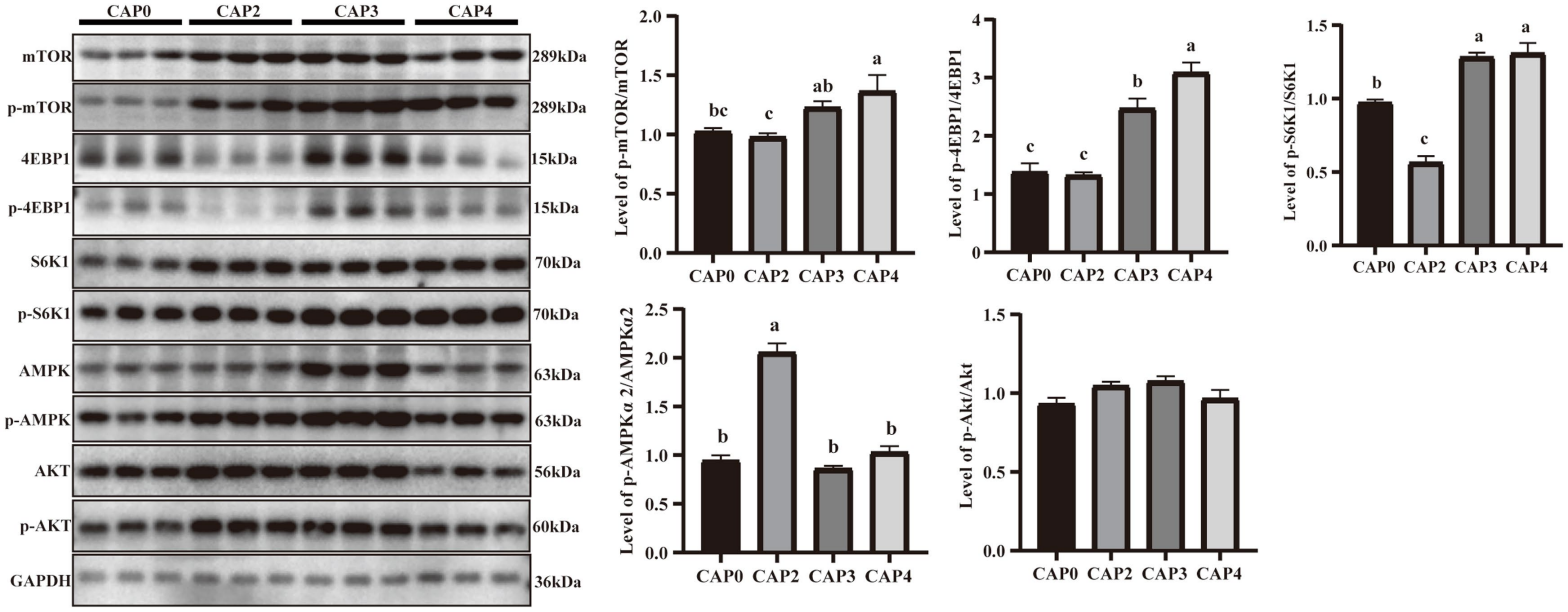
Protein expression levels and phosphorylation levels of some selected liver genes involved in protein synthesis and energy metabolism in broilers fed CAP diets. Data are presented as means and standard errors (±SE) (*n* = 6). Significant differences (*p* < 0.05) are indicated by different alphabetical superscripts above the bars, while the same letter indicates no difference.

Figure 4 presents the protein phosphorylation level of key regulators in mTOR signaling compared between the different treatments in the liver. The mTOR, 4EBP1, and S6K1 phosphorylation levels in the CAP3 and CAP4 groups were higher than the CAP0 and CAP2 groups (*p <* 0.05). The phosphorylation level of AMPKɑ2 was higher in the CAP2 group than in the other groups (*p <* 0.05).

## Discussion

There is currently limited research on CAP in livestock and poultry, with most studies focusing on aquatic animals. Several studies have demonstrated that an appropriate proportion of CAP can enhance animal growth and development, improve production performance, and promote good health (26, 27, 30, 31). However, a high proportion of CAP substitution may lead to a decline in animal production performance (32, 33), as well as intestinal and liver damage, and increased mortality (15). Wu et al. (34) found that adding the proper amount of CAP in broiler diets improved growth rate and feed conversion efficiency, which is consistent with our findings. Therefore, the function of CAP may be related to the ease of digestion (15, 34).

Assessing the slaughtering performance is crucial in determining the quality of livestock and poultry carcasses. Therefore, it is important to consider the breast and leg muscle yields as they are crucial indicators of meat production performance. Previous research has shown that supplying an appropriate level of SCP in broiler diets can improve carcass quality and increase breast and leg muscle yields (14), which is consistent with the results of this study. Another study reported that the supplement of different proportions of SCP in broiler diets did not affect the dressing percentage, which aligns with the findings of the present study (35). According to the study, including CAP in the diet of broilers increased the CP content in muscle, suggesting an acceleration of protein synthesis and deposition. These findings are consistent with previous studies conducted by Xue et al. (28) and Wu et al. (10) in aquatic animals.

Serum biochemical indicators change according to the body’s health and metabolic status. Measuring these indicators can visually reflect the body’s health, nutrition, and metabolic status. TP reflects the state of protein absorption and synthesis, and high TP content indicates vigorous protein metabolism, which can enhance the body’s immune function and promote growth and development (36, 37). ALB plays a crucial role in stabilizing blood osmolality and transporting metabolic substances (38). UA is a protein metabolite produced mainly in the liver and excreted through the kidneys and intestines. Hyperuricemia and gout can be caused by overproduction or reduced excretion of UA (39). Previous study has shown that the inclusion of CAP in diets can increase TP and ALB levels, and UA levels also tend to increase with increasing CAP content (34), which is consistent with the results of the present study. TC and TG are the main lipids in the blood and important indicators of lipid absorption, metabolism, and transport (40, 41). In the present study, CAP had a significant effect on the lipid metabolism of broilers, as evidenced by the decrease in TG levels. This finding is consistent with the results reported by Wu et al. (34) but differs from those observed in aquatic animals (11, 27), which may be attributed to species differences.

AST and ALT are often used as indicators of liver injury (42). ALP acts as a dephosphorylator and has an important biological function, commonly used to assess liver injury and disease (43–45). It is worth noting that ALP levels increase during skeletal development when the organism is growing rapidly (46). In the present study, AST and ALP tended to increase when adding CAP to the diet. It was suggested that CAP may cause some degree of damage to liver function while promoting rapid growth of the organism. Consistent with the results of the present study, previous studies have shown an increasing trend in AST with increasing CAP (15, 33). Meanwhile, Maulu et al. (31) showed that 5 and 10% CAP increased ALP activity, which is similar to the results of the present study.

SOD reduces oxidative damage by scavenging free radicals and generating oxygen molecules and hydrogen peroxide. T-AOC reflects the body’s total antioxidant capacity, and GSH-Px converts harmful peroxides into harmless hydroxyl compounds, protecting the normal structure and function of cell membranes (47). In the present study, including CAP in the diet increased GSH-Px and SOD activities, as well as T-AOC, indicating that the appropriate proportion of CAP can enhance the organism’s antioxidant capacity. This finding is consistent with previous studies that have shown CAP’s ability to increase antioxidant enzyme activities and improve the organism’s antioxidant capacity (30, 31, 34). Conversely, a high proportion of CAP may impair the organism’s antioxidant defense system, leading to oxidative damage (11, 17). Therefore, we speculate that this situation could be due to substitution ratio, species differences, and the absence of specific nutrients required by certain animals in CAP. For instance, it was reported that taurine deficiency results in elevated levels of oxidative damage in fish, which has a detrimental impact on health and economic performance. (48).

IgG is the main Ig in the organism, accounting for about 75% of the total amount of serum Ig (49), and IgA is the second most abundant, accounting for 10–20% of the total serum Ig (50). Previous studies have shown that CAP can upregulate immune-related indicators and enhance immune function, which is consistent with the results of the present study (18, 26, 31). IL-1, IL-6, and IL-8 are members of the interleukin family and play a crucial role in regulating inflammation and immune responses in the body (51, 52). Maulu et al. (31) reported that CAP did not have any adverse effects on the regulation of inflammation and immune responses, as IL-8 levels were not significantly different from the control group. Similarly, the present study found no significant differences in IL-8 levels among the groups, indicating that CAP did not cause an inflammatory response in the body.

The mTOR pathway integrates various external signals from energy, amino acids, inflammatory factors, and hormones, with a vital function in nutrient sensing and regulation (53). Additionally, it plays a significant role in protein deposition, growth and development, and metabolic regulation in the body (54). Animal weight gain is primarily due to the deposition of muscle and fat, wherein muscle deposition primarily depends on protein synthesis, and accelerating protein synthesis can promote rapid organism growth (10, 55). Numerous studies have demonstrated that the genes *mTOR*, *4E-BP1*, and *S6K* were associated with protein synthesis. Activation of mTOR was typically accompanied by changes in the expression of *4E-BP1* and *S6K* genes, but *4E-BP1* plays a more significant role in protein synthesis than *S6K* (10, 27, 56, 57).

Previous studies have shown that an appropriate proportion of CAP can enhance the expression of genes associated with the mTOR signaling pathway, stimulate muscle protein synthesis, and enhance the organism’s growth performance (10, 27, 28). Furthermore, Li et al. (26) also found that the dietary inclusion of CAP improved the expression levels of genes related to growth, antioxidants, and the TOR pathway. The present study showed that the *Lipin-1* gene was down-regulated, and the *AMPKɑ2*, *Akt*, and *4E-BP1* genes were up-regulated compared to the control group, suggesting that the appropriate proportion of CAP activates the mTOR signaling pathway and promotes the organism’s growth and development, which is consistent with previous studies.

The phosphorylation levels of S6K1 and 4E-BP1 are typically indicators of the degree of activation of the mTOR pathway, and activation of the mTOR pathway can promote cell proliferation and growth (56, 58, 59). In the present study, the CAP3 group significantly increased the levels of phosphorylation of 4E-BP1, S6K1, Akt, and AMPKɑ2, which provides further evidence at the protein expression level that a suitable proportion of CAP can activate the mTOR signaling pathway and regulate organism growth. Consistent with the results of this study, Maulu et al. (27) reported that the phosphorylation levels of 4E-BP1, S6K1, and AMPK significantly increased by 5 and 10% CAP. However, in contrast to the results of our study, some researchers found that adding CAP to the diet inhibits the mTOR pathway and negatively affects the organism’s growth and development (60, 61). The discrepancies in these studies may be due to species specificity and variations in the amount of substitution, and further investigation is needed to determine the exact reasons.

The activation of the mTOR pathway closely links to amino acid (AA) levels. It has been proven that AAs, particularly branched-chain amino acids (BCAAs), regulate protein synthesis through the mTOR pathway by controlling *S6K1* and *4E-BP1* activities (62, 63). Leucine, the primary BCAA that regulates protein synthesis, accelerates protein synthesis in the organism by activating the mTOR pathway, thereby promoting growth (20, 64, 65). Therefore, we speculate that the growth-promoting effect of CAP may be related to its high AA content and digestibility (15, 66). However, it is worth noting that CAP has a low content of arginine and a high content of nucleic acids. Arginine is an important regulator of the mTOR pathway, and a lack of arginine leads to a decrease in protein synthesis, which may explain why a high proportion of CAP negatively affects organismal growth and development.

## Conclusion

In this study, adding 3% CAP to broiler diets reduced the FCR (22–42 d) and increased the breast and leg muscle yields as well as the CP content of chicken meat. Adding 3% CAP to broiler diets also improved the antioxidant properties (T-AOC, GSH-Px, and SOD) and enhanced the IgG of the organism. In addition, the inclusion of 3% CAP to the diet activated the mTOR pathway and increased the levels of 4E-BP1, S6K1, Akt, and AMPKɑ2 phosphorylation. Notably, the addition of 4% CAP to the diet increased serum UA, AST, and ALP. Based on the above results, our recommended use level in broiler diets is 3%.

## Data availability statement

The datasets presented in this study can be found in online repositories. The names of the repository/repositories and accession number(s) can be found in the article/Supplementary material.

## Ethics statement

The animal studies were approved by Laboratory Animal Welfare Guidelines of China. The studies were conducted in accordance with the local legislation and institutional requirements. Written informed consent was obtained from the owners for the participation of their animals in this study.

## Author contributions

CS: Writing – original draft, Visualization, Validation, Methodology, Investigation, Formal analysis, Data curation, Conceptualization. YL: Writing – original draft, Validation, Software, Methodology, Formal analysis, Conceptualization. CM: Writing – original draft, Validation, Resources, Methodology. CL: Writing – original draft, Software, Resources, Methodology. QL: Writing – original draft, Validation, Resources, Methodology, Investigation. SL: Writing – original draft, Software, Methodology, Formal analysis, Data curation. GJ: Writing – review & editing, Visualization, Validation, Supervision, Project administration, Funding acquisition, Conceptualization. JT: Writing – review & editing, Visualization, Validation, Supervision, Project administration, Funding acquisition, Conceptualization.
